# Exploring the Role of Peer Health Navigators in the Australian Health System: A Qualitative Interview Study

**DOI:** 10.1111/hex.70453

**Published:** 2025-10-08

**Authors:** Natali Cvetanovska, Simone Said, Rebecca L. Jessup

**Affiliations:** ^1^ Staying Well and Hospital Without Walls Program Northern Health Epping Australia; ^2^ School of Rural Health Monash University Warragul Australia; ^3^ School of Allied Health, Human Services and Sport La Trobe University Melbourne Australia

**Keywords:** equity, health access, health literacy, health systems, lived experience, navigation, peer health navigators

## Abstract

**Background:**

Peer health navigators (PHNs) work with their own communities to provide health navigation support and connect with patients through shared experience and identity. The aim of this study was to explore the role of PHNs in the Australian health system from the perspective of managers and experts, with a focus on understanding how to build and sustain this workforce in Australia.

**Methods:**

Individuals who employ and manage PHNs, as well as subject matter experts in the navigation field from a range of health and academic organisations across Australia, participated in semi‐structured interviews. A descriptive qualitative approach based on naturalistic inquiry was used to analyse the interview data. Themes were framed around an adapted socio‐ecological model using four levels: individual, interpersonal, organisational and societal (combined community and policy).

**Results:**

Seventeen themes were identified within the four levels of the adapted socio‐ecological model: individual (personal attributes, knowledge and skills, lived experience), interpersonal (boundary setting: professional, boundary setting: professional meets personal, boundary setting: self‐care, supervision and debriefing), organisational (recruitment, qualifications and prerequisites, clear job description and expectations, training, role duties and tasks) and societal (value of peer navigators in Australia, government support, funding, strategic approach to workforce planning, workforce governance).

**Conclusion:**

PHNs are a valuable part of the Australian health system. A cohesive, strategic, national approach is required to ensure the development of the workforce and the sustainability of programs and allow for navigators to be embedded within the Australian health system.

**Patient or Public Contribution:**

This study is part of a larger project that developed formal education and training courseware materials for health navigators in Australia. The materials were co‐designed with patients, managers and subject matter experts. In this paper, we sought the perspective from managers and experts across Australia who work with diverse groups of patients.

## 1 Background

Peer health navigators (PHNs) help patients navigate health services and remove barriers to accessing care. They work across various health conditions and settings [1, 2, 3] and, at the core of the role, they assist patients by providing education and linking patients to services and resources [4]. Organisations implement health navigator programs to support patients with complex health needs, improve access to services and healthcare, reduce hospitalisation, improve screening and improve health outcomes for patients [5]. Implemented navigator programs have shown multiple benefits in reducing health disparities [6], improving access to care and improving health outcomes [4].

Health navigators come from various backgrounds and can be health professionals (often nurses or social workers) or lay people with no clinical qualifications [2]. We have used the term ‘peer health navigators’ to refer to health navigators without qualifications, as they work with their own communities to provide health navigation support [7] and can connect with patients in a way that health professionals may not be able to, through the use of shared experience and identity [8]. PHNs can be especially beneficial in engaging culturally and linguistically diverse (CALD) communities, communities that have a distrust of healthcare services and other underserved populations [8].

The Australian health system is not one system but a connection of multiple services and funders, which makes it difficult for patients to understand and navigate [9]. A PHN workforce is a potential solution to some of this complexity; however, this is a newer health workforce in Australia with less support and fewer governance structures compared to traditional health workforce roles. Recently, a number of peak bodies in Australia have recommended the implementation of PHN roles to address system access issues and reduce health disparities [10, 11, 12]; these recommendations could help steer the PHN conversation in Australia to help build up the PHN workforce and to get the required government support. There is a long history of the PHN role in the United States [13]. While we can learn from the US experience, the implementation of PHNs must be context‐specific [4]. Therefore, the aim of this study was to explore the role of PHNs in the Australian health system from the perspective of managers and experts, with a focus on understanding how to build and sustain this workforce in Australia.

## 2 Materials and Methods

### 2.1 Study Design

We conducted a qualitative study using semi‐structured interviews investigating the perspectives of managers and subject matter experts (SMEs) on the role of PHNs and PHN programs in Australia. We did not include perspectives of PHNs, patients or health professionals in this article; however, the authors sought these perspectives in another study [14].

### 2.2 Participants and Sample

Opportunistic sampling was used to recruit individuals who employ and manage PHNs as well as SMEs in the navigation field. Participants came from a range of health and academic organisations across Australia. Participants were known to the research team and were directly invited to participate by a member of the research team. We aimed to interview a range of managers and SMEs, working with various patient populations and in different settings, to ensure that different areas of navigator expertise were covered.

### 2.3 Ethical Considerations

The study was approved by the Northern Health Low Risk Ethics Committee (Project ID: 87830).

### 2.4 Procedure and Data Collection

Data were collected using semi‐structured interviews. Question areas were developed by N.C., S.S. and R.L.J. and focused on: the value of the PHN workforce in Australia, the barriers and enablers to the successful implementation of navigator programs, and the skills and training of PHNs. Participants were individually interviewed by N.C. Informed consent was gained before participation. Interviews were conducted on Microsoft Teams, were recorded and transcribed verbatim.

### 2.5 Theoretical Framework

Data analysis was framed around the socio‐ecological model [15]. This model comprises five levels: individual, interpersonal, organisational, community and policy. For this study, we adapted the model by combining community and policy into one level: societal. We did this to reflect the broader social influences that impact health navigation, such as cultural norms, public awareness and policy frameworks, which together shape both community interactions and the regulatory environment. This adaptation allowed us to capture both the direct and indirect societal factors influencing the role and effectiveness of PHNs within the Australian health system.

### 2.6 Data Analysis

A descriptive qualitative approach based on naturalistic inquiry [16, 17] was used for the data analysis to enable us to provide an observation that accurately reflects the experiences and perspectives of participants. We used the socio‐ecological model levels as parent codes. To ensure that themes match the content of the interviews, two researchers (N.C. and R.L.J.) independently conducted a first round of deductive coding of a sample of one‐third of the transcripts using NVivo software (QSR International). N.C. is a health services researcher with no clinical qualifications, and R.L.J. is an experienced qualitative researcher and clinician. The researchers then met to compare codes, combine similar codes and determine a list of sub‐themes as they applied to the socio‐ecological model levels. N.C. then coded all transcripts against the final list of sub‐themes. Both researchers met again to finalise the data analysis.

### 2.7 Data Reporting

This study followed the Standards for Reporting Qualitative Research [18] to ensure transparency and completeness of reporting.

## 3 Results

### 3.1 Participant Characteristics

Seventeen participants took part in an individual interview; the interview duration was a mean of 37 min. Nine participants are managers of PHNs, and eight are SMEs in the peer navigation field. Participants represented diversity across sectors, including metropolitan health services (*n* = 5), community health and primary care services (*n* = 5), universities (*n* = 4), a regional health service (*n* = 1), a not‐for‐profit (*n* = 1) and a private health insurer (*n* = 1). Together, they represented diverse expertise in peer navigation across areas that included aged care, chronic disease management, cancer services, CALD communities, disability, LGBTQIA+ and mental health.

### 3.2 Themes Arising From Data Analysis

A total of 17 sub‐themes were identified within the levels of the socio‐ecological model, as detailed in Table 1.

**Table 1:** Themes emerging from interviews.

Socio‐ecological level	Sub‐theme
Individual	Personal attributes Knowledge and skills Lived experience
Interpersonal	Boundary setting: Professional Boundary setting: Professional meets personal Boundary setting: Self‐care Supervision and debriefing
Organisational	Recruitment Qualifications and prerequisites Clear job description and expectations Training Role duties and tasks
Societal	Value of PHNs in Australia Government support Funding Strategic approach to workforce planning Workforce governance

#### 3.2.1 Individual

##### 3.2.1.1 Personal Attributes

Participants outlined the ideal personal attributes for individuals employed in PHN roles. When managers are recruiting for PHN roles, these are the characteristics they look for in applicants at an interview. All participants pointed to the importance of PHNs being strong communicators who are able to build relationships. This included being a good listener, being able to communicate with all people, being able to relate to people, being respectful, showing compassion and empathy, being approachable and enjoying interacting with people. In their interactions, it was identified that it is important to be genuine and authentic to build trust and rapport.

‘I think they need to have heart and be people who can have … tough conversations with people and able to … quite quickly build rapport so that they can have that sort of honest and quite personal conversation’.

Participant 9, SME

Other ideal personality traits included dynamic and committed individuals who are able to cope with uncertainty, are resourceful and good problem solvers, have a collaborative mindset and ability to work in a team, and are curious and eager to learn. Some participants who manage PHNs also identified that PHNs need to be mentally tough and robust to be able to support others who are experiencing difficulty or suffering.

‘A level of robustness to be able to support someone who's going to be on a daily basis talking to you about their distressing experiences [and their] suffering’.

Participant 7, Manager

Together, these perspectives emphasise that PHNs require strong interpersonal skills and emotional resilience, enabling them to connect meaningfully with clients while sustaining their own well‐being in demanding roles.

##### 3.2.1.2 Knowledge and Skills

Participants discussed the knowledge and skills individuals need to bring to PHN roles. Knowledge and skills relate to the tacit knowledge an individual must possess to succeed in their role and differ from personal attributes, which are the characteristics and traits of an individual. For those working in bicultural PHN roles, knowing the language and understanding the culture of the community they are working in was deemed essential.

‘For bilingual navigators it's critical to understand the language, understand culture, understand the beliefs of those communities’.

Participant 16, SME

However, the English skills of the PHN must also be strong to accurately complete documentation and communicate effectively within the organisation they work for—they are acting as a conduit or bridge between the two.

‘We need a reasonably high level of English literacy … all of our communication and things are in English so we need people to be able to read and interpret that, and to then feedback to community’.

Participant 5, Manager

Further skills and knowledge required of PHNs are having a good level of health literacy, the ability to set up projects and skills in building partnerships with the community. An important consideration that came up frequently in the interviews was computer literacy. The increasing reliance on digital tools within the Australian healthcare environment means that PHNs must be able to use the tools and systems provided to them by their organisation.

‘We have started to check for a basic level of computer literacy because we do need that in order to navigate our systems, and because they'll have to record data’.

Participant 5, Manager

Overall, participants highlighted that PHNs need a broad foundation of cultural, linguistic, health and digital literacy skills, enabling them to operate effectively as trusted bridges between their communities and the health system.

##### 3.2.1.3 Lived Experience

Another individual factor identified was lived experience, which was seen as an essential attribute for the PHN role due to the importance of the relational nature of the role in establishing connections with communities. Typically, PHNs should have a shared lived experience for the population they are working with. This could be having the same health condition, having an experience of being a refugee or asylum seeker, or sharing a culture and/or language, membership of a community centred around identity (e.g., LGBTQIA+, disability and neurodiverse communities, interfaith and religious minority communities) or lived experience as a carer. It was seen as important that anyone applying for PHN roles must understand that, and they must have lived experience.

‘We have started to come up with different strategies around making sure that in our advertisements, it's really clear, “You must have a lived experience. Do not apply unless you have a personal lived experience.” And even before we offer people interviews we double check, “Just want to make sure this is a lived role and you're aware of that.”’

Participant 7, Manager

Participants emphasised that shared lived experience is a core attribute required for the PHN role, as it fosters trust, authenticity and meaningful connections with the communities they serve.

#### 3.2.2 Interpersonal

##### 3.2.2.1 Boundary Setting: Professional

The participants who manage PHNs felt that it was important for PHNs to be able to set professional boundaries by understanding what is and is not within the PHN role and ensuring that others (clients and colleagues) understand that too. Professional boundary issues may arise directly when PHNs do not understand the limitations of their roles, or may occur when health professionals or patients do not fully understand the role and may ask PHNs to do tasks that are outside of scope. It was seen as important that PHNs feel comfortable saying when something is not within their role.

‘The other thing is you can also set boundaries about your role and you're clear when you can say “No, I can't help you.”… So, one of the real skills is saying no’.

Participant 8, SME

An important method to ensure boundaries are maintained is to have clear guidelines available that describe the tasks PHNs can and cannot do.

‘Something that we really push is we have a diagram that says “This is what peer workers do, and this is what peer workers don't do.” So, it's really clear … peer workers don't write referrals they don't do assessments’.

Participant 7, Manager

Overall, participants highlighted that maintaining clear professional boundaries, supported by explicit role guidelines, is essential to protect PHNs, clarify expectations and ensure the role is understood by both colleagues and clients.

##### 3.2.2.2 Boundary Setting: Professional Meets Personal

Being part of the community in which they work can raise some other boundary issues for PHNs. PHNs are serving their own communities, often within small community groups. This may mean that PHNs are living within the community but are also working with these same people in the health context.

‘Another boundary to highlight here is boundary between community and professional life. So, that's a really tricky one. So, like, I guess when it's a marginalised community … [LGBTQIA+] community in particular, like we don't stop being who we are when we stop working. And that's just the reality of it and as much as peer navs can, for example, hand over a client that they might have a first person or second‐degree connection with sometimes [it] can be unavoidable. So suddenly the person who you see at the parties now is your intake worker and seeing all this personal information. So that's a very, I think significant boundary that it's really hard to hold’.

Participant 14, Manager

A solution for this particular issue is having large teams or teams with people outside of the community, where PHNs can refer the client to someone else who is not involved with the client.

‘That's why we have employed a community health nurse and their manager is not a local resident so they've got an ability to say, “Hey, this one is too close to home” or “I can't take on that one … remove that responsibility from me.”’

Participant 5, Manager

This sub‐theme highlights the difficulty of maintaining boundaries when PHNs live and work within the same community. Having another worker in the organisation who is not part of the community can help PHNs to navigate this challenge.

##### 3.2.2.3 Boundary Setting: Self‐Care

It was also seen as important than PHNs are able to manage personal boundaries and practice self‐care. The nature of PHN roles is that PHNs are engaging with their own communities and with people that have similar lived experiences, which can be re‐traumatising to the PHN.

‘They pay a high tax because it is your own trauma that you're reliving. So, if you're a refugee, you get a refugee client coming in, they don't just talk about their health, they talk about what happened 5 years ago. They talked about what happened last week when they were buying groceries, and they heard this, loud noise or watched the news, and they saw something. So, the re‐traumatisation aspect … we just need to be very mindful of, how do we look after people?’

Participant 14, Manager

This sub‐theme highlights that PHNs must be supported to maintain personal boundaries and engage in self‐care, as working closely with communities that mirror their own lived experiences can be emotionally demanding and, at times, may be re‐traumatising.

##### 3.2.2.4 Supervision and Debriefing

Many participants recognised the importance of having someone to talk to should any boundary issue arise. Supervision and debriefing are seen as critical to help PHNs determine boundaries and discuss issues as they arise. Being part of a team and having good support structures within the team are essential.

‘Every time that they'd finish a shift [they] would have a 15 min debrief and in that they would … talk about their experience and what happened and … sometimes when they were overstepping their boundaries, they just needed to be reminded’.

Participant 11, Manager

Structured supervision, regular debriefing and strong team support are important to helping PHNs navigate boundary challenges and sustain their well‐being in the role.

#### 3.2.3 Organisational

##### 3.2.3.1 Recruitment

The managers of PHNs said that they found it easy to recruit for PHN roles. They often have large candidate pools with many suitable applicants who are keen to work within their communities.

‘But what we've since learned with the recruitment is it's not difficult to get [applicants] … that's not a challenge in most communities. It's not a challenge to get interest in the roles’.

Participant 5, Manager

The final issue was that many people who would be suitable for PHN roles did not know that these types of roles exist.

‘I think just people first and foremost actually knowing that these roles exist. I just think that there's really poor awareness out there…. We need to come up with more strategies around how do we attract people to the workforce…. I think it's really hard for people to know that it exists’.

Participant 7, Manager

Managers reported that while PHN roles attract strong interest and are generally easy to recruit for, broader community awareness of these roles remains limited, highlighting the need for targeted strategies to reach potential candidates.

##### 3.2.3.2 Qualifications and Prerequisites

Many participants felt that PHNs do not need a baseline certification or qualification; the only prerequisites required for a PHN role are the individual qualities and knowledge described above.

‘Our navigators [need] to have shown a demonstrated connection to the community that we're working [in] … because we wanted that lived experience and we also prioritised like the top languages that were spoken. So, all of our navigators, it's a requirement that they speak at least one other language and a priority language in the community, so they were prioritised over qualifications because we recognised that's actually what was missing in our work is actually the connection’.

Participant 5, Manager

It was highlighted that PHNs often originate from marginalised groups and may already face many barriers to employment; therefore, making a qualification mandatory might create an unnecessary barrier to employment.

‘Say that you have to have the certification, that would be a massive disservice to the community because when marginalised communities are marginalised in one area, such as health, they often also experience marginalisation in social life, with their family supports, with their networks, with access to economic participation…. So, the person who is successful usually is coming through these barriers and overcoming these barriers, so we would never want to put yet another barrier’.

Participant 14, Manager

A number of participants agreed that formal qualifications are not required for PHN roles, as lived experience, community connection and language skills are more important to the role. Making certification mandatory might create unnecessary barriers for candidates from underserved communities.

##### 3.2.3.3 Clear Job Description and Objectives

Most participants felt that PHNs would benefit from a well‐defined role description that made their role responsibilities clear. However, the participants who are managers also acknowledged that their staff often had unclear role definitions. Some PHN roles were developed from existing PHN models, while others evolved organically based on the needs of patients or communities at the time. These participants shared their desire for these roles to have more structure.

‘Have a very clear aim for those roles and a very clear set of outcomes that they would like to see those roles achieving’.

Participant 6, SME

Performance indicators were also seen as a way for PHNs to know how success in their role was measured, giving them clear goals to work towards and for managers to have clear measures to evaluate the success of PHNs.

‘Having a structure around the program and … the objectives [is important]. This includes what we're trying to achieve and having documentation to show that and be able to monitor it’.

Participant 11, Manager

Participants emphasised that clear role definitions, structured objectives and measurable outcomes are essential to guide PHNs, support their managers and provide a framework for evaluating success.

##### 3.2.3.4 Training

Training provided to PHNs varied widely. Some participants who were managers provided their PHNs with structured training programs at commencement, as well as ongoing development opportunities. Others described a more informal approach to training through shadowing others and being given feedback as the need arises.

The training topics described by participants across the interviews included mental health first aid, quality improvement, boundary setting, confidentiality, cultural awareness, LGBTQIA+ awareness, bicultural worker training, workplace health and safety, and peer support training. Much of the training was delivered by external organisations, and managers allowed PHNs to pursue professional development in the areas in which each individual PHN was interested.

‘[Navigators] have access to professional development programs so they can actually undertake courses that they seek, but we don't standardise those courses…. So, if they are more interested in mental health we develop them in mental health. If they are more interested in medical administration, we encourage them to do so as long as everyone can actually cover the core requirements, then the I guess flair they bring is welcome and we don't try to standardise our people’.

Participant 14, Manager

The way training was delivered was also varied among participants. Some PHNs were provided with training manuals and others were given the opportunity to role‐play different scenarios, while others experienced a combination of both. Training was also delivered via case studies, conferences and events, communities of practice and/or internally developed presentations.

‘It takes about a week of talking, a good solid week of talking and a lot of practicing, quite often, I roleplay patients with the new [navigators], as have others. And then a lot of practice, it takes about 4–8 weeks to become pretty comfortable in your skin doing it’.

Participant 4, Manager

Some challenges managers faced in training PHNs are that places are often limited in external training programs and that organisational budgets do not cover professional development for PHNs.

‘We get zero training budget for our workforce … so any training I offer to our team all has to be free or we either have to apply for a grant or try to raise money for it’.

Participant 7, Manager

Participants emphasised that clear role definitions, structured objectives and measurable outcomes are important to guide PHNs, support their managers and provide a framework for evaluating success within the roles.

##### 3.2.3.5 Role Duties and Tasks

While there were some minor variations in the description of tasks performed by PHNs, the majority of tasks remained consistent across roles. Many tasks involved the PHNs interacting directly with patients and their families, including speaking to patients after they have engaged with the health organisation, helping them to understand information that they have been given, attending appointments at a patient's request, visiting patients on wards, and acting as a point of contact and facilitating support groups. Other tasks were finding or developing resources for patients and connecting with external organisations.

‘They meet with patients and carers and families. They'll meet with them, provide support, provide them with advice … and they would take them to appointments, they'd visit them in the wards, they'd visit them in the waiting rooms. They also run or co‐facilitate the support groups’.

Participant 11, Manager

Tasks also went beyond just direct patient interactions. PHNs often engage with the community to build trust. They also provided education to communities. For example, one participant spoke about PHNs engaging their communities during the Covid‐19 pandemic with information about vaccines.

‘People were in the testing sites, or in the vaccination sites to help community feel that safe environment, understand what type of vaccine [they are being given]’.

Participant 12, Manager

Some participants also described PHNs providing education to staff within their organisation on their particular area of expertise.

‘I think the main tasks are really building staff capability [for example] “Staff aren't using pronouns correctly, can you come and talk to them?” [or] “We've got this person coming in, it didn't go very well last time, can you come and talk to the team about how we can get ready and do things better?”’

Participant 15, Manager

The tasks of PHNs span direct patient and family support, community engagement and staff education, reflecting a multifaceted role that bridges gaps between patients, health services and the broader community.

#### 3.2.4 Societal

##### 3.2.4.1 Value of PHNs in Australia

All participants agreed that PHNs bring value to the Australian health system and to society. PHNs are especially important in the context of a fragmented system that can be difficult for patients to navigate, where they face many barriers to care. PHNs can help patients access services that they may not be aware exist. Participants also identified that PHNs can play a role in health equity, helping vulnerable groups to access services, helping with transitions from one setting to another and navigating the health system generally.

‘Health navigators are not just required, they're essential. While Australia has a great healthcare system, it's a complex health system and it's multiple systems within systems’.

Participant 1, SME

Many participants stated that most people would benefit from some form of navigation support. However, most commonly, they reported that navigation may be particularly beneficial for people with complex health needs who need to access multiple providers. Being from a culturally or linguistically diverse background can add a layer of complexity to accessing services and engaging with the health system, and because of this, participants felt this was another group that could benefit from PHNs.

‘[Navigators are] for everyone, but I think for particular persons that that have multi morbidities, so multiple conditions, multiple chronic conditions, but also if you overlay that people that have English is not the first language, so that have cultural, linguistic, other I guess, let's say domains that within their lives that make it more challenging for them’.

Participant 1, SME

Participants also acknowledged that while PHNs could bridge the gap between patients and services, they couldn't necessarily eliminate it. While PHNs do their best, they are limited by the health system and by what services exist.

‘We're making health care more and more complicated for people to actually get around…. So, people often give up before they get to what they might really need’.

Participant 10, Manager

Participants agreed that PHNs add significant value by helping people, particularly those with complex needs or from diverse backgrounds, navigate a fragmented health system and improve equity in access to care.

##### 3.2.4.2 Government Support

Many participants agreed that the government needs to provide the funding, resources and policies required to establish and embed the PHN workforce. With government support, the PHN workforce could be better coordinated across the fragmented and complex health system.

‘It's good to look at the Aboriginal health workforce health workers as an example. In Australia, so Aboriginal health workers have been there for a long time and they actually have a significant contribution to Aboriginal health … we've got that example in Australia because there is at the top level … that political commitment for that’.

Participant 16, SME

Participants underscored that sustained government commitment through funding, resources and policy is critical to embed and strengthen the PHN workforce across the health system.

##### 3.2.4.3 Funding

Funding was a challenge consistently raised by participants. Programs are often funded on a short‐term basis or through grants for pilot programs. When the funding ends, so do the PHN programs. Some funding is short‐term and not guaranteed, creating uncertainty. For participants who employed PHNs funded through grants, even if it was the same grant they had applied for in the past, often application requirements and funding amounts changed, so it became difficult for them to plan for these roles in the longer term.

‘[Funding is] yearly and … each time it changes and requirement and amount of money, everything changes’.

Participant 12, Manager

As the funding was not ongoing or the organisation was uncertain whether the funding would be renewed, PHNs often left the roles to look for permanent or more secure work elsewhere. Many participants identified that without secure funding, it was difficult to keep staff, and this provided an ongoing challenge to the sustainability of the program.

‘The fact that [the role is] contracted … it's not a permanent position, so therefore, there's always going to be an issue coming into the end of a contract of where whether a person's willing to stay or move on to something else, if something more permanent comes up’.

Participant 10, Manager

Short‐term and uncertain funding was seen as a key barrier to workforce stability, with participants linking secure, ongoing investment to staff retention and program sustainability.

##### 3.2.4.4 Strategic Approach to Workforce Planning

An issue identified by many participants was that navigation programs in Australia have not developed in a strategic way, leading to silos and duplication of efforts in developing and implementing programs and adding more complexity to an already complex health system.

‘And one of the things that does concern me is the silos…. You've got all of these different levels of government, both state and Commonwealth, who were funding all of these programs and … they overlap, they duplicate…. You look at the [recommendations from the] Disability Royal Commission, you look at the [National Disability Insurance Scheme] review, you look at the Aged Care Royal Commission, you look at the Productivity Commission into early childhood, you look at the state Mental Health Royal Commission … they're all recognising that people are getting lost in the systems and finding it really hard to get the right services. But is the best solution having all of these [different] pockets of money…? Is this really the best way to support people?’

Participant 17, Manager

One suggestion for a more strategic approach was the development of a national model and credentialing framework that healthcare providers could potentially adopt when employing PHNs. Although PHNs operate in diverse contexts, establishing a set of baseline principles would be beneficial, allowing each setting or condition to adapt the model to meet its specific needs.

‘I think having a national framework and national model that could guide each organisation, each population group to develop their own [PHN] program would be really helpful … it doesn't mean that everybody has to follow the same model. It depends on the needs of the population group’.

Participant 16, SME

Participants called for a more coordinated, national approach to PHN workforce planning to reduce duplication, enhance consistency and ensure that programs effectively meet population needs.

##### 3.2.4.5 Workforce Governance

Some participants suggested that a national model for education and a competency framework to assess skills in the workplace could provide governance support, set a scope of practice, give the role legitimacy and status, and help the public to understand the navigator role. Participants also stated that a national education framework would be useful for the navigator workforce. A structured education framework would allow for common baseline knowledge, skills and competencies to be defined and would allow for professional development.

‘That would be great to establish … an education system, certain accreditation or credentialing system … to be able to get educated within a framework and certain system to be able to set up a proper workforce that each [organisation] can recruit from’.

Participant 2, SME

A national governance framework could also be useful in establishing clear employment conditions. This could include formalising the role within a health enterprise bargaining agreement and setting pay rates and working conditions. It could also involve establishing employment tiers and pathways to allow career progression for navigators.

‘[Navigators] can then start lobbying for some sort of enterprise bargaining … because they've got so many different employers and they're often in not‐for‐profit organisations and that makes it difficult’.

Participant 13, SME

Overall, a national framework for education, competencies and employment conditions was seen as essential to provide legitimacy, enable career progression and establish a sustainable PHN workforce.

## 4 Discussion

Our study provides new insights into the role of PHNs in Australia from the perspective of the managers who employ PHNs as well as academic experts in the field. We categorised findings using the socio‐ecological model, identifying themes across individual, interpersonal, organisational and societal levels, with 17 sub‐themes emerging.

There was a strong consensus among study participants that PHNs are a valuable part of the Australian health system and may address health disparities and improve health outcomes for underserved populations. PHNs can reduce barriers to care, enhance health literacy and support care transitions to improve health outcomes and health equity [19]. The role is especially valuable in the context of a complex health system. This is also a recurring theme in the international literature; health systems around the world are increasingly fragmented and difficult to navigate for patients and their families, and this particularly impacts those who are most vulnerable [20, 21]. Our findings on the importance of lived experience as a core attribute for PHNs align with international literature. Studies highlight the important role of shared lived experiences in building trust and rapport, especially among CALD communities and those who experience issues with trust in the healthcare system [7]. Similarly, research has shown that PHNs with shared cultural or experiential backgrounds improve access to care and adherence to treatment plans among Indigenous populations and immigrants [20]. Our findings support these observations, particularly in the Australian context, where just over half of the population are first‐ or second‐generation migrants [22].

The requirement for PHNs to have lived experience means that managing professional and personal boundaries can be complex, as they do not have a structured scope of practice [23] nor do they have the same level of training to handle boundaries as healthcare professionals [19], so knowing where the boundary is may be difficult. Our findings indicate that PHNs also often live in the same community in which they work and may encounter their patients at events, have friends in common or congregate at the same place of worship. Internationally, studies have recognised that the blurred lines between personal and professional relationships are a unique challenge for PHNs as they form close bonds with patients and their community [24, 25]. The need for structured training and regular supervision and support to address these challenges is echoed in the international literature [19, 23, 26]. Organisations should provide the opportunity for PHNs to debrief with a senior staff member regularly and encourage them to engage in self‐care behaviours.

Participants who managed PHNs reported that the roles appear to be attractive, and recruiting to PHN roles is generally straightforward. They often experience large candidate pools with many suitable applicants who are keen to work within their communities. The fact that there are no prerequisite education requirements or other qualifications can make these roles attractive to those who want to help people but do not have a clinical qualification. Once recruited, it is important for PHNs to have a clear job description to understand their duties and responsibilities, as well as performance indicators to measure their success in performing these duties. Structured training can help PHNs understand their role and how to perform their duties. However, participants stated that they often do not have clear job descriptions for their PHNs and tasks are decided on an ad hoc basis, and training is often inadequate and also on an ad hoc basis. Moreover, similar to our findings, international studies found that a lack of standardisation in training and role descriptions creates ambiguity around PHN roles, hindering their integration into healthcare systems [27]. Furthermore, despite working across different conditions and settings, we found there was consistency in the tasks PHNs do, indicating that standardised core competencies and training in Australia would be useful for PHNs.

The challenges we identified in establishing and maintaining PHN roles in Australia mirror those found internationally. Funding instability has been identified as a key limitation in navigation programs globally, with short‐term grants often leading to program discontinuation and loss of workforce momentum [4, 28]. Ongoing, reliable sources of funding are a major threat to sustainability in Australia. Healthcare funding is complex, and multiple funding sources and payers exist in Australia [9, 29]. This makes it unlikely that there will be one source of funding for PHNs, and they will likely be funded differently depending on where in the system they are working. This will include a mix of federal, state and private funding across different care settings, including hospitals, community organisations, social care organisations, services operating under the national disability insurance scheme or private health. Many interview participants stated that their navigator programs were funded through short‐term grants, so programs are often abandoned once the funding ends. This means that staff leave looking for other work, and so all the work that has gone into these programs is wasted.

The planning and governance of the PHN workforce has been another challenge. Workforce planning has been uncoordinated and has not taken a strategic approach, again leading to sustainability issues and duplication of efforts. To achieve sustainable and successful navigator programs, these issues must be addressed [4]. Given the similarity of tasks that PHNs are doing, this new workforce could benefit from a national model of credentialing to standardise the scope of practice, core competencies, skills and knowledge requirements for PHNs and allow flexibility for context‐specific requirements. Other research has also found that an overarching national educational approach would help to establish core competencies and standardise skills and knowledge requirements [26, 27]. Furthermore, clear employment conditions like those in an enterprise bargaining agreement could be useful to add structure to PHN roles, outline clear career pathways and provide wage information. Some PHN roles are unpaid and staffed by volunteers. While some volunteers are happy to share their experience for free, this may be unfair to the people in the roles and can undervalue the important work of PHNs [8].

Our study's strength lies in its inclusion of participants encompassing diverse levels of expertise, working with various types of PHNs, and serving a wide range of populations. Across this diverse group, we identified many themes, which were systematically organised using the socio‐ecological model to capture the multilevel factors influencing PHN roles. Our study also had a number of limitations. The reliance on self‐reported data from managers and SMEs introduces the potential for bias, as these perspectives may not fully capture the lived experiences of PHNs or the patients they serve. Furthermore, the study's focus on the Australian context may limit the applicability of findings to health systems with differing structures and cultural dynamics.

## 5 Conclusion

PHNs are a valuable part of the Australian health system. A cohesive, strategic, national approach is required to ensure the development of the workforce, the sustainability of programs and allow for PHNs to be embedded within the Australian health system. This may help patients to access healthcare and potentially improve health outcomes for all Australians, especially those from underserved communities.

## Author Contributions

**Natali Cvetanovska:** conceptualisation, methodology, formal analysis, investigation, writing – original draft. **Simone Said:** conceptualisation, methodology, writing – review and editing. **Rebecca L. Jessup:** conceptualisation, methodology, formal analysis, writing – review and editing.

## Ethics Statement

The study was approved by the Northern Health Low Risk Ethics Committee (Project ID: 87830).

## Conflicts of Interest

The authors declare no conflicts of interest.

## Data Availability

The data that support the findings of this study are available from the corresponding author upon reasonable request.
